# Membrane Fixation and Graft Persistence in Pre-Extraction Buccal Overbuilding: A Split-Mouth Histological Study in Dogs

**DOI:** 10.3390/dj14090547

**Published:** 2026-09-01

**Authors:** Takahisa Iida, Shunsuke Baba, Giovanna Iezzi, Ermenegildo Federico De Rossi, Fernando Maria Muñoz Guzon, Karol Alí Apaza Alccayhuaman, Daniele Botticelli

**Affiliations:** 1 Department of Oral Implantology, School of Dentistry, Osaka Dental University, 8-1 Kuzuhahanazonocho, Hirakata, Osaka 573-1121, Japan; iidataka@iris.ocn.ne.jp (T.I.); baba-s@cc.osaka-dent.ac.jp (S.B.); 2 Department of Medical, Oral and Biotechnological Sciences, University of Chieti-Pescara, 66013 Chieti, Italy; gio.iezzi@unich.it; 3 ARDEC Academy, 47923 Rimini, Italy; e.f.derossi@gmail.com; 4 Ibonelab, Centro de Empresas e Innovación Nodus, 27003 Lugo, Spain; fernandom.munoz@usc.es; 5 Department of Veterinary Clinical Sciences, Facultade de Veterinaria, Terra Campus, Universidade de Santiago de Compostela, 27002 Lugo, Spain; 6 Biomaterials and Technology, Department Research, University Center for Dental Medicine Basel UZB, University of Basel, 4058 Basel, Switzerland; caroline7_k@hotmail.com

**Keywords:** pre-extraction grafting, buccal overbuilding, membrane fixation, collagen membrane, titanium pins, bovine bone mineral, graft stabilization, histomorphometry, dog model, buccal contour

## Abstract

**Background:** Buccal overbuilding may support the buccal contour before tooth extraction, but the effect of collagen membrane fixation on graft stability remains unclear. This study evaluated whether pin fixation influences buccal ridge dimensions and graft persistence in a pre-extraction dog model. **Methods:** In six Beagle dogs, bovine bone mineral was grafted bilaterally at the mandibular first molar region without tooth extraction and covered with a native collagen membrane, fixed with titanium pins at test sites and left unfixed at control sites. After three months, histological measurements were performed. The primary outcome was buccal bone width at 2 mm apical to the cemento-enamel junction. **Results:** Buccal bone width was 1.02 ± 0.30 mm in the Pin group and 0.89 ± 0.37 mm in the No-pin group. The mean paired difference was 0.13 mm (95% CI: −0.55 to 0.81 mm; *p* = 0.6875). The combined outer mineralized contour measured 1.06 ± 0.37 mm and 0.95 ± 0.42 mm, respectively. Residual graft occupied 19.4 ± 6.9% of the standardized buccal region in the Pin group and 8.7 ± 6.6% in the No-pin group. **Conclusions:** Membrane fixation did not significantly improve buccal bone width after three months. The greater residual-graft fraction in the Pin group was exploratory and requires confirmation in studies including tooth extraction and subsequent implant placement.

## 1. Introduction

Following tooth extraction, the alveolar socket undergoes a complex sequence of healing and remodeling events aimed at replacing the blood clot with newly formed bone and progressively restoring tissue continuity within the socket [1,2,3]. However, this process is invariably associated with dimensional changes of the alveolar ridge, particularly affecting the buccal aspect, where the bone wall is often thin and more susceptible to resorption [4,5]. Several experimental and clinical studies have shown that neither spontaneous healing nor immediate implant placement can fully prevent this remodeling process, which may compromise ridge morphology and the long-term stability of peri-implant tissues [6,7,8,9,10,11].

Beyond the structural characteristics of the buccal bone wall, recent biological models have suggested that post-extraction ridge reduction may also be influenced by osteoclastic activity on the external surface of the alveolar bone, where it interfaces with the oral mucosal barrier [12]. In contrast to the coupled bone remodeling occurring within the extraction socket, these so-called oral barrier osteoclasts may not be followed by equivalent osteoblastic bone formation, resulting in a net loss of alveolar bone structure. Local mucosal injury, inflammatory responses, mechanical stimulation, and connective tissue contraction have also been proposed as potential contributors to this osteoclastogenic environment.

Based on these anatomical and biological considerations, several ridge preservation and guided bone regeneration procedures have been proposed to reduce post-extraction dimensional changes. These approaches include the use of bone substitutes with or without collagen membranes [13,14,15], dense PTFE membranes [16,17,18], cortical laminae [19,20,21,22,23,24], the socket-shield technique [25,26,27], growth factors [28,29,30], tooth-derived grafts [31,32,33,34], and other regenerative strategies aimed at supporting socket healing and preserving the buccal contour [35,36,37].

Juxta-extraction [38,39,40,41,42,43] and pre-extraction [44,45] buccal overbuilding procedures have also been investigated as alternative strategies aimed at supporting the buccal contour. However, the available preclinical evidence suggests that these procedures may not be sufficient to prevent post-extraction buccal bone resorption, despite maintaining an augmented compartment containing biomaterial and newly formed tissue.

Nevertheless, the potential relevance of such an augmented compartment should not be interpreted exclusively in terms of coronal ridge enlargement. In selected clinical situations, the regional distribution of the grafted material may also be important, since cervical and apical portions of the buccal ridge may have different biological and prosthetic implications. Therefore, evaluating not only the coronal buccal profile but also the persistence and distribution of residual graft particles may provide useful information on the behavior of pre-extraction buccal overbuilding procedures.

Collagen membranes are widely used in guided bone regeneration and alveolar ridge preservation because of their biocompatibility, chemotactic properties, favorable tissue integration, and ability to act as a temporary barrier against soft-tissue cell invasion. However, collagen membranes differ in their degradation kinetics and mechanical properties according to tissue origin and structural organization. Their limited intrinsic space-maintaining capacity, particularly after hydration, may reduce their ability to stabilize particulate grafts and resist soft-tissue pressure in non-contained defects [46,47,48,49]. In these conditions, the mechanical stability of the membrane remains a relevant issue. Membrane displacement may compromise graft stability, reduce space maintenance, and influence the pattern of new bone formation. Some studies have suggested that membrane fixation may enhance bone regeneration or improve graft containment, whereas others have reported limited or no additional benefit, especially in contained defects or when flap design and suturing provide sufficient stabilization [50,51,52,53].

In a recent preclinical study on post-extraction defects with immediate implant placement, fixation of a collagen membrane with pins did not significantly improve vertical bone gain or osseointegration compared with a non-fixed membrane [54]. However, membrane fixation appeared to limit the displacement of residual graft particles, suggesting a possible role in maintaining the position of the biomaterial during healing.

Therefore, the present study was designed to investigate whether fixation of a collagen membrane with pins could influence membrane stability, graft containment, and the regional distribution of a buccal graft in a pre-extraction buccal overbuilding model, before the onset of extraction-related remodeling processes. Particular attention was given to the coronal/cervical buccal profile, while the persistence and distribution of residual graft particles in more apical regions were also evaluated to better understand the behavior of the augmented compartment.

## 2. Materials and Methods

### 2.1. Ethical Statement

The animal experiment was conducted in compliance with European Directive 2010/63/EU, which regulates the protection of animals used for scientific purposes. Ethical approval was obtained from the Ethical Committee of the Rof Codina Foundation and the competent regional authority, Xunta de Galicia, Xefatura Territorial da Consellería do Medio Rural, Ronda da Muralla 70, Lugo, Spain, under authorization code 01/20/LU-001, dated 8 April 2020. The study followed the ARRIVE reporting guidelines, and the experimental procedures were initiated in June 2023.

### 2.2. Experimental Animals

The study included six adult female Beagle dogs, approximately six years old, with a mean body weight of 11.5 kg. The animals were obtained from Isoquimen, Barcelona, Spain, and were kept under quarantine and allowed to acclimatize for three weeks before the beginning of the experimental phase. Housing, husbandry, and experimental procedures were conducted at the CeBioVet research facility in Lugo, Spain. All animals were considered clinically healthy after the quarantine period. The animals were not genetically modified. No previous experimental procedures were reported.

### 2.3. Experimental Design

The present investigation was designed as a prospective, randomized, split-mouth controlled animal study. Buccal grafting was performed bilaterally in the mandibular first molar region without tooth extraction. A particulate graft was placed on the buccal aspect of the distal root and covered with a collagen membrane. On the test side, the membrane was fixed with pins placed at its mesial and distal margins, whereas the membrane was left unfixed on the contralateral control side. Both experimental sites were treated during the same surgical session in each animal. Healing was allowed to proceed for three months before histological evaluation.

The three-month endpoint was selected a priori to evaluate the early-to-intermediate phase of healing. This period was considered sufficient to allow initial bone formation and incorporation of the graft particles, while still permitting the assessment of potential differences in graft retention and dimensional stability related to membrane fixation. The selected observation period was also consistent with previous preclinical dog studies using comparable regenerative procedures [45,54] and broadly corresponded to the interval during which substantial degradation of pericardium-derived collagen membranes has been reported [55]. The study was not intended to assess complete long-term remodeling of the slowly resorbing xenograft.

Six dogs were included, each contributing one Pin site and one contralateral No-pin site, resulting in six paired observations and six sites per experimental condition. The dog was considered the experimental unit.

### 2.4. Sample Size Calculation and Methodological Rationale

In strict accordance with the 3Rs principles (Replacement, Reduction and Refinement) for animal experimentation, a split-mouth design was selected. This approach minimizes inter-individual biological variance by allowing each animal to serve as its own internal control, thereby reducing the total number of animals required to achieve statistical validity compared with a parallel-group design.

The rationale for using the biomaterial in combination with a collagen membrane was primarily to support the coronal/cervical portion of the buccal ridge, where dimensional preservation after tooth extraction is clinically relevant. However, the concept of pre-extraction buccal overbuilding may not be limited to coronal ridge augmentation alone. Increasing the buccal thickness in more apical regions may also be of interest, particularly in clinical situations where future implant placement is prosthetically driven and the implant axis may bring the apical portion of the implant close to the buccal alveolar wall. Accordingly, measurements in the more apical regions were included to characterize the regional distribution and persistence of the grafted compartment. No clinical benefit related to subsequent implant placement could be assessed in the present model. Therefore, while the coronal/cervical region remained the primary area of interest, the apical distribution and persistence of residual graft particles were also evaluated as exploratory findings related to the regional behavior of the augmented compartment.

Bone thickness measured at 2 mm apical to the cemento-enamel junction (CEJ) was selected a priori as the primary outcome variable. The CEJ provided a stable and reproducible anatomical reference. In a previous clinical study on immediate implant placement, the thickness of the buccal and lingual/palatal bone walls was assessed approximately 1 mm apical to the alveolar bone crest [8]. In the present study, the alveolar crest was located, on average, approximately 0.6–0.7 mm apical to the CEJ; therefore, the 2-mm CEJ level corresponded approximately to a level 1.3–1.4 mm apical to the bone crest. This measurement consequently represented the coronal portion of the buccal ridge while avoiding the most coronal supracrestal connective tissue attachment area. The other horizontal measurements, including those recorded in more apical regions, as well as the distance from the CEJ to the most coronal aspect of the alveolar crest, were regarded as exploratory descriptive variables aimed at characterizing the regional behavior of the grafted compartment.

Based on the previous dental literature evaluating dimensional changes around immediately placed implants, where implant position provided a reproducible reference for horizontal measurements, reduced buccal bone thickness has been associated with greater horizontal ridge reduction after tooth extraction [10]. Therefore, a clinically relevant paired difference in bone thickness between the two experimental conditions was defined as δ = 0.5 mm. Assuming a standard deviation of the within-animal paired differences of σd = 0.33 mm, this corresponded to a standardized paired effect size of Cohen’s dz = 1.52. The required sample size was calculated using G*Power software v. 3.1 under the “Means: Difference between two dependent means (matched pairs)” option. With a two-sided α level of 0.05 and a statistical power of 80%, six animals were required. With six paired observations, the estimated statistical power was approximately 84%. The paired *t*-test framework was used only for sample-size planning because the anticipated effect was expressed as a mean paired difference and its standard deviation. The final inferential analysis was performed using the Wilcoxon matched-pairs signed-rank test, as described in Section 2.13.

### 2.5. Randomization, Allocation Concealment, and Blinding Procedures

Randomization of test and control sites was performed electronically by one author (D.B.), who did not participate in the surgical procedures. Allocations were coded and stored in sealed opaque envelopes until surgery. The assigned treatment was revealed only after flap elevation and preparation of the recipient area.

Histological slides were anonymized before microscopic evaluation. As fixation pins were not identifiable in the histological sections, treatment allocation could not be identified during the assessment. Measurements were performed following predefined standardized histometric protocols.

The split-mouth design was used to minimize inter-animal variability, as each dog received both experimental conditions. Random allocation of the Pin and No-pin treatments to the contralateral mandibular sites was used to minimize potential side-related confounding. No additional strategies were used to control potential confounders related to animal housing or the order of histological measurements.

The author who generated the randomization sequence (D.B.) was aware of treatment allocation. The surgeon became aware of the assigned treatment only after flap elevation and preparation of the recipient area. The histological evaluator (E.F.D.R.) remained blinded to group allocation throughout outcome assessment. Group allocation was disclosed for the statistical analysis after completion of the histological measurements and finalization of the dataset.

### 2.6. Biomaterial Characteristics

Cerabone^®^ granules 0.5–1.0 mm (Botiss biomaterials GmbH, Zossen, Germany) were used as filler material. Cerabone^®^ is a xenogeneic bovine bone mineral produced from cancellous bone through high-temperature processing at approximately 1200 °C. This treatment removes the organic components and results in a ceramic hydroxyapatite scaffold with a porous architecture. The material is characterized by high macroporosity, with pore sizes reported to range approximately from 100 to 1500 μm, a rough hydrophilic surface, osteoconductive properties, and low resorption rate, contributing to the long-term stability of the grafted area.

Jason^®^ membrane (Botiss biomaterials GmbH, Zossen, Germany) was used to cover the grafted area. It is a native collagen membrane derived from porcine pericardium and composed mainly of collagen type I, with a lower proportion of collagen type III. The membrane is not chemically cross-linked and preserves the natural three-dimensional collagen structure of the pericardium.

### 2.7. Anesthesia Procedures

Before surgery, the animals were premedicated intramuscularly with medetomidine 10 μg/kg (Sededorm^®^ 1 mg/mL, Vetpharma Animal Health, Barcelona, Spain) combined with morphine 0.3 mg/kg (Morfina Braun 2%; B. Braun Medical, Barcelona, Spain). General anesthesia was induced by intravenous administration of propofol 2 mg/kg (Propofol Lipuro^®^ 10 mg/mL, B. Braun, Melsungen, Germany). After induction, anesthesia was maintained with 1–1.5% isoflurane delivered in oxygen (Vetflurane^®^ 1000 mg/g, Virbac SA, Carros, France).

During the anesthetic procedure, the animals were continuously monitored for heart rate, respiratory rate, arterial blood pressure, and CO_2_ levels. These parameters were recorded every 5 min during the initial phase after infusion onset and every 15 min thereafter.

### 2.8. Surgical Procedures

**Figure 1:**
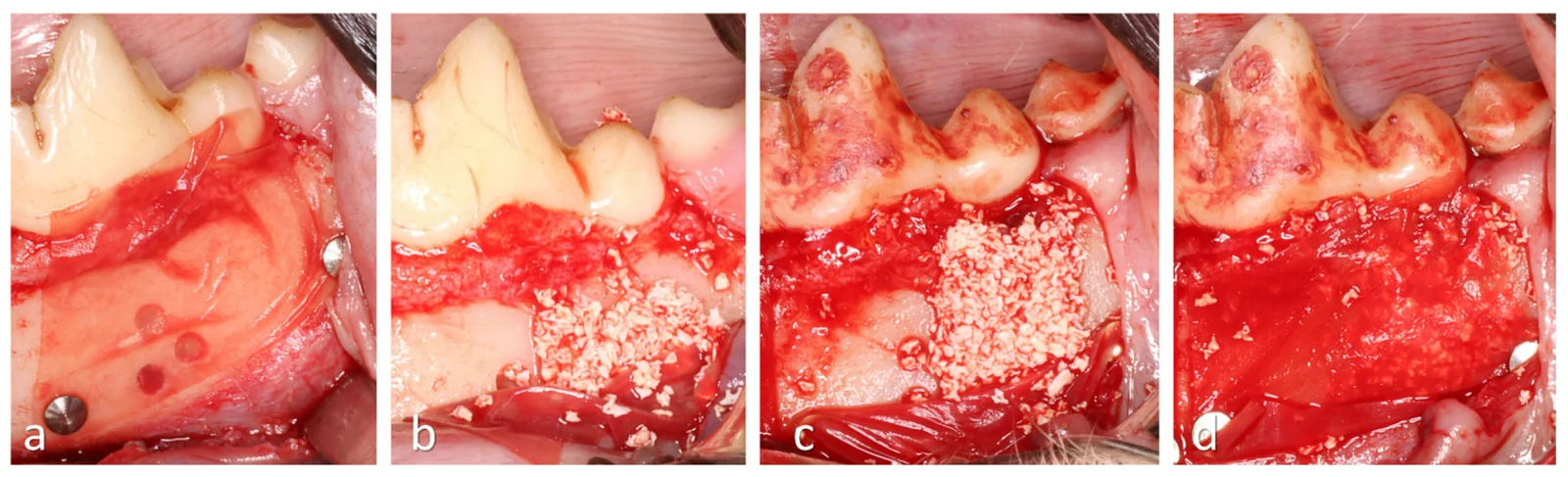
Surgical procedure in the Pin group. (**a**) A Jason collagen membrane was adapted to the buccal aspect of the treated area and secured with two titanium pins. (**b**,**c**) The membrane was temporarily reflected apically, and Cerabone^®^ granules were placed for buccal overbuilding. The graft was shaped around the coronal aspect of the bone crest while also extending over the more apical buccal surface. (**d**) The membrane was repositioned over the grafted area before flap closure.

A full-thickness flap was elevated bilaterally in the mandibular first molar region and extended apically to expose the buccal bone surface overlying the distal root. The cortical bone was gently abraded with a curette. Three perforations were then prepared approximately in the interradicular region using a 2-mm round bur. The bur was advanced through the buccal cortical plate until a decrease in resistance was perceived and then inserted further into the underlying bone. The same recipient-bed preparation was performed in both experimental conditions. A Jason collagen membrane was adapted to cover the buccal aspect of the treated area. In the Pin group, the membrane was secured with two 3-mm-long titanium pins (titan pin set; Botiss Biomaterials GmbH, Zossen, Germany), placed through the membrane and anchored into the underlying buccal cortical bone. One pin was positioned at the mesial and one at the distal margin of the grafted area, apical to the alveolar crest. Each pin was engaged by the tip of the dedicated pen-type applicator and driven into the cortical bone using a surgical mallet until its entire penetrating portion was embedded within the cortical bone and the pin head was seated against the membrane, thereby stabilizing the membrane against the recipient surface. The position and insertion trajectory were selected according to the local root anatomy to avoid contact with or damage to the roots (Figure 1a).

The membrane was then temporarily reflected apically, Cerabone^®^ granules were placed for buccal overbuilding (Figure 1b,c), and the membrane was repositioned over the grafted area (Figure 1d). The flaps were closed with interrupted Vicryl 4-0 sutures. In the No-pin group, the same procedure was performed, except that the membrane was not fixed with pins.

### 2.9. Housing and Husbandry

For the first two weeks, plaque control was carried out three times weekly by wiping the teeth with gauze moistened with 0.12% chlorhexidine. After this initial period, the animals underwent toothbrushing three times per week with chlorhexidine gel until completion of the study.

Pain control was achieved using buprenorphine 0.01 mg/kg intramuscularly every 8 h (Bupaq, Richter Pharma AG, Wels, Austria). Meloxicam (Loxicom, Norbrook Laboratories, Monaghan, Ireland) was administered intravenously before surgery at 0.2 mg/kg and then orally for the following two days at 0.1 mg/kg once daily. During each surgical intervention, antibiotic prophylaxis consisted of cefazolin 20 mg/kg administered intravenously (Cefazolina Normon, Madrid, Spain). In addition, cefovecin sodium 8 mg/kg was administered subcutaneously as a single long-acting injection (Convenia^®^, Zoetis, Madrid, Spain).

### 2.10. Euthanasia

After three months of healing from the final surgical procedure, the animals were sedated and subsequently euthanized by intravenous administration of an overdose of sodium pentobarbital at 200 mg/kg (Dolethal, Vetoquinol, Magny-Vernois, France).

### 2.11. Histological Preparation

The harvested tissue blocks were fixed in 10% buffered formaldehyde, dehydrated through ascending concentrations of ethanol, and embedded in light-curing resin (Technovit 7200 VLC; Heraeus-Kulzer GmbH, Wehrheim, Germany). Radiographic images were obtained to identify the root canal spaces of the distal and mesial roots and to guide buccolingual sectioning through the region of interest. The blocks were then sectioned using a precision band saw system (Exakt Apparatebau, Norderstedt, Germany). The sections were progressively ground and polished to obtain histological specimens with a final thickness of approximately 50 µm. The slices were stained with acid fuchsin and toluidine blue before the analysis.

### 2.12. Histological Evaluations

Histological slides were examined using an Eclipse microscope (Nikon Corporation, Tokyo, Japan) connected to a digital image analysis system. Measurements were obtained using NIS-Elements D 5.11.00 software (Laboratory Imaging, Nikon Corporation, Tokyo, Japan).

All measurements were performed at the distal root, corresponding to the area where the biomaterial was applied and covered by the collagen membrane, either fixed with pins at the test sites or left unfixed at the control sites.

**Figure 2:**
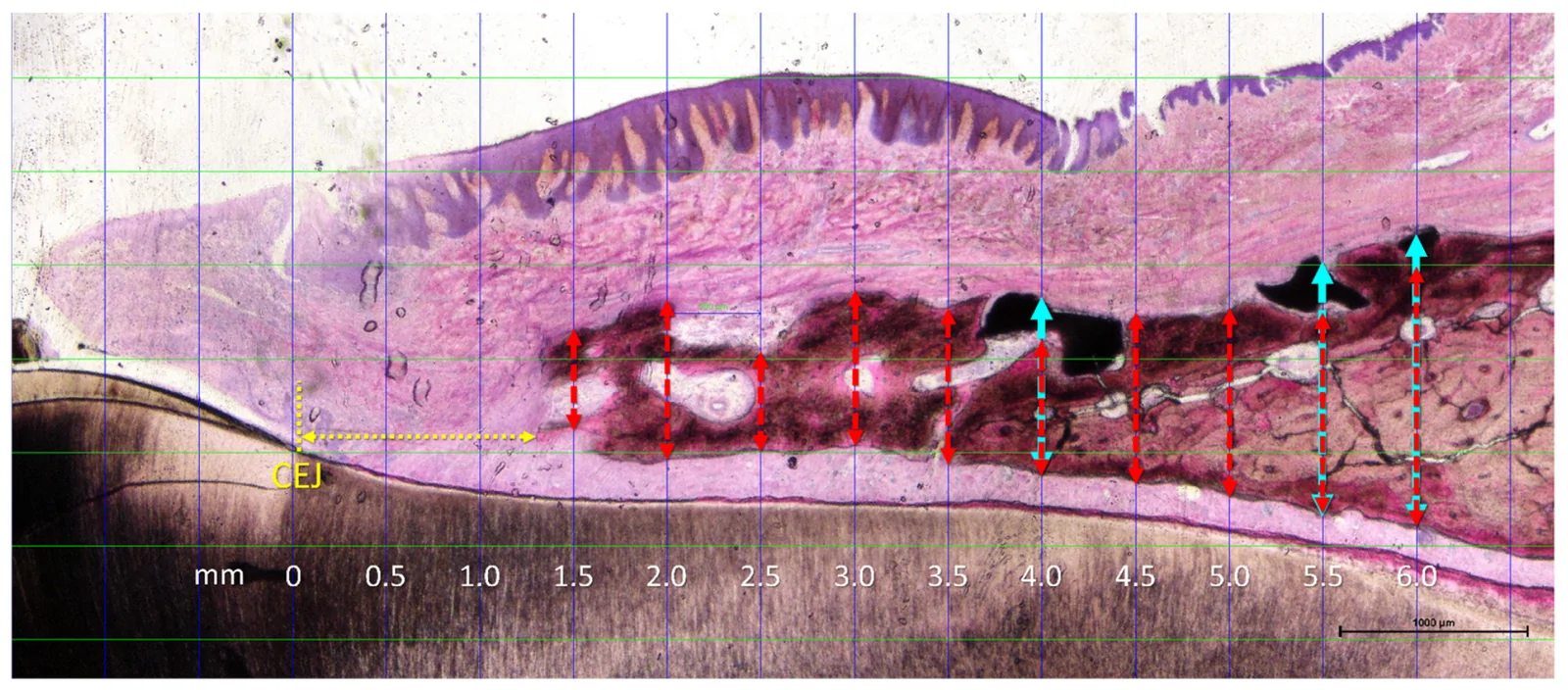
Measurement method. The buccal alveolar crest width was measured at consecutive 0.5-mm intervals, from the cementoenamel junction (CEJ) to 6 mm apically, using a lattice superimposed on the histological image. At each level, bone width was measured from the inner margin of the alveolar crest to the outer bone contour (red dashed arrows). A combined outer mineralized contour was also evaluated by considering residual graft particles when they extended beyond the outer bone contour (light blue dashed arrows). The yellow dotted line indicate the distance between CEJ and the top of the bone crest.

The buccal bone width was measured at consecutive 0.5-mm intervals, from the cementoenamel junction (CEJ) to 6 mm apically, using a lattice superimposed on the histological image (Figure 2). At each level, bone width was measured from the inner margin of the alveolar crest to the outer bone contour. A combined outer mineralized contour was also calculated to account for residual graft particles when they extended beyond the outer bone contour.

The vertical distance from the CEJ to the alveolar bone crest was recorded (Figure 2). In addition, the most coronal level of the combined outer mineralized contour was identified as the most coronal mineralized point represented by either bone or residual graft particles.

An additional exploratory histomorphometric analysis was performed within a standardized buccal region of interest using the same CEJ-based reference system illustrated in Figure 2. The region extended from the coronal margin of the alveolar bone crest to the horizontal line located 6 mm apical to the CEJ. It was delimited internally by the bone contour facing the periodontal tissues and externally by the combined outer mineralized contour. Within this region, the areas occupied by bone, residual graft particles, and non-mineralized tissue were delineated and expressed as percentages of the total analyzed area. Non-mineralized tissue included all tissue not classified as bone or residual graft and consisted predominantly of connective tissue surrounding the graft particles, with only occasional limited marrow spaces.

### 2.13. Data Analysis

All histological measurements were performed by one evaluator (E.F.D.R.) after calibration with an experienced examiner (D.B.). Since all measurements were linear, intra-examiner reproducibility was assessed using the intraclass correlation coefficient (ICC) on a subset of randomly selected sections measured twice after a two-week interval. The intra-examiner agreement was excellent, with ICC values above 0.90 for all linear measurements.

The dog was considered the experimental statistical unit. No additional a priori inclusion or exclusion criteria were established for animals, experimental units, histological sections, or individual data points. All six animals completed the study, and no animals, experimental sites, histological sections, or data points were excluded from the analyses. Each analysis therefore included six paired observations, with one Pin site and one No-pin site contributed by each dog. Descriptive statistics were calculated as means and standard deviations for each variable and experimental condition.

The prespecified primary outcome variable was the horizontal buccal bone width measured 2 mm apical to the CEJ. This was the only outcome subjected to formal inferential testing.

The combined outer mineralized contour measured at the same level was considered a secondary descriptive outcome. The horizontal bone width and combined outer mineralized contour measured at all other levels, as well as the vertical distance from the CEJ to the most coronal level of the alveolar bone crest and to the most coronal level of the combined outer mineralized contour, were considered exploratory descriptive variables aimed at characterizing the regional behavior of the grafted compartment. These variables were summarized as means and standard deviations and were not subjected to formal hypothesis testing.

The percentages of bone, residual graft, and non-mineralized tissue within the standardized buccal region of interest were considered additional exploratory outcomes and were summarized as means and standard deviations without formal hypothesis testing.

Because the study followed a split-mouth design and included a limited number of paired observations, the primary outcome was compared between the Pin and No-pin sites using the Wilcoxon matched-pairs signed-rank test. Formal normality testing was not used because of the low power of normality tests in small samples. Since only one prespecified primary comparison was subjected to inferential testing, no adjustment for multiple comparisons was required. The level of significance was set at *p* < 0.05. Statistical analyses were performed using GraphPad Prism, version 11.0 (GraphPad Software, San Diego, CA, USA).

## 3. Results

### 3.1. Clinical Evaluations

No adverse events occurred during the healing period. All biopsy specimens were available for histological evaluation.

### 3.2. Qualitative Histological Evaluation

**Figure 3:**
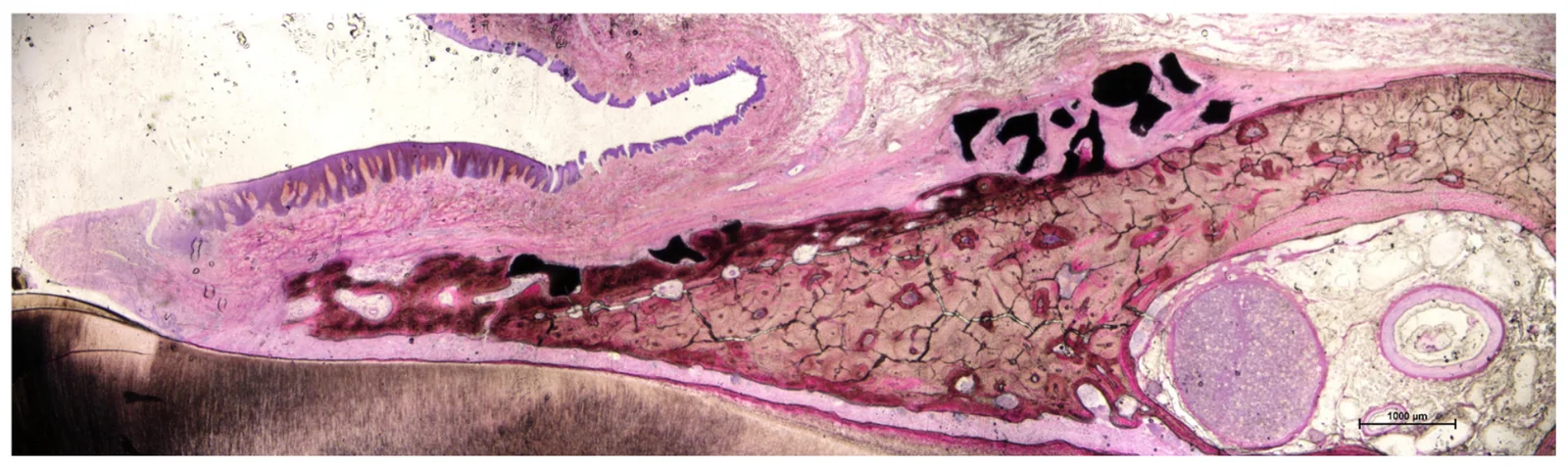
Photomicrograph of a ground section from the Pin group. The buccal bone crest appeared to be positioned within an anatomically normal range in relation to the CEJ. Bone remodeling was evident at the coronal and buccal aspects of the crest. Residual graft particles were visible, with partial integration into newly formed bone. Acid fuchsin and toluidine blue stain.

In both groups, the buccal bone crest appeared to be positioned within an anatomically normal range in relation to the CEJ, although areas of bone remodeling were observed, particularly in the Pin group (Figure 3).

**Figure 4:**
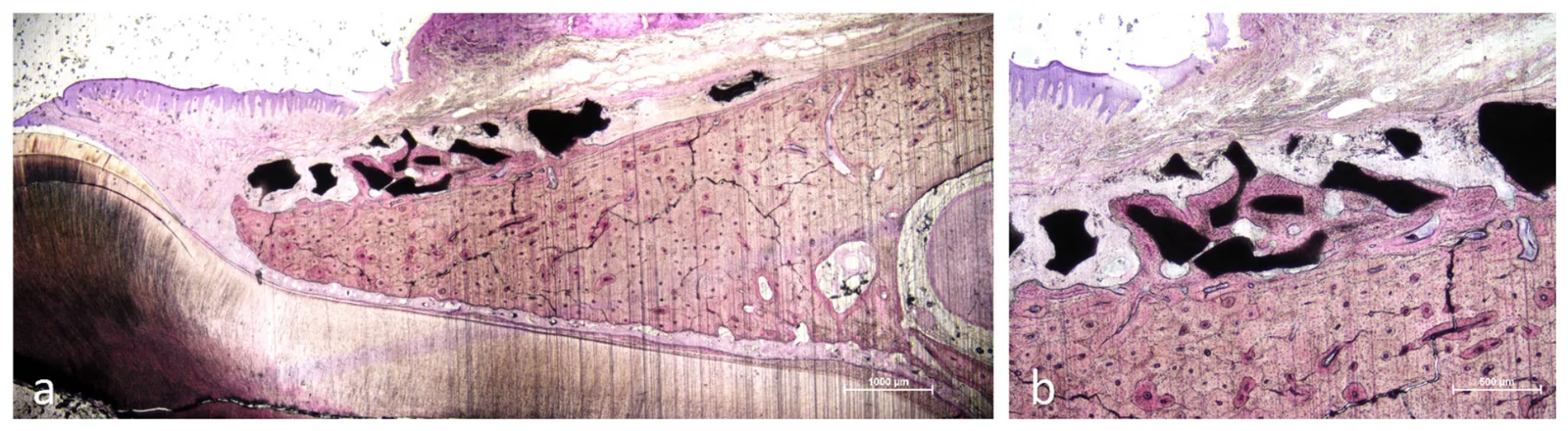
Photomicrographs of a ground section from the Pin group. (**a**) In a few cases, residual graft particles were observed in a coronal position. (**b**) Higher magnification of the area shown in (**a**), illustrating residual graft particles incorporated within newly formed bone. Acid fuchsin and toluidine blue stain.

In the Pin group, few specimens showed residual graft particles located coronally, close to the buccal aspect of the alveolar bone crest (Figure 4a).

**Figure 5:**
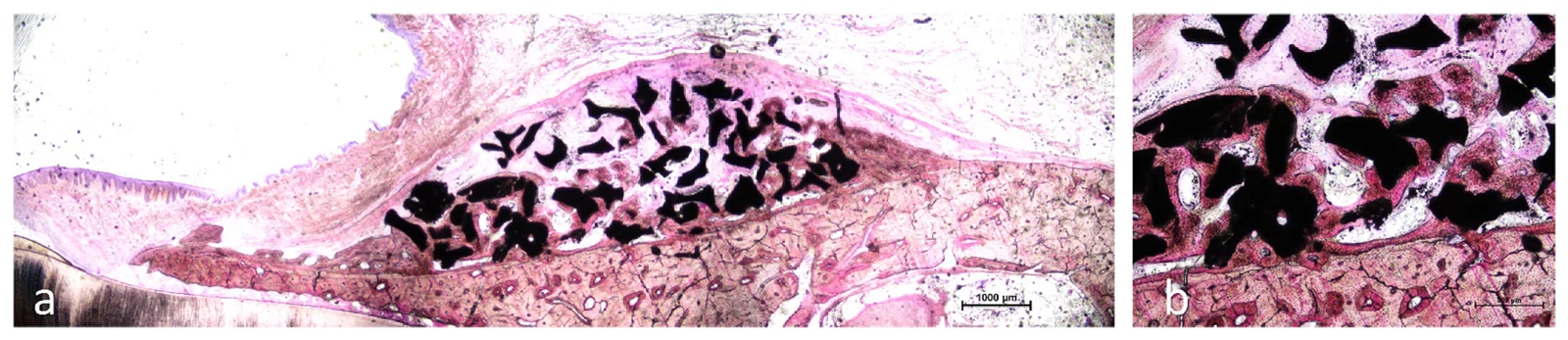
Photomicrographs of a ground section from the Pin group. (**a**) Residual graft particles were frequently located in more apical regions, in some cases beyond 6 mm from the CEJ, 1000 μm. (**b**) Higher magnification of the area shown in (**a**), showing residual graft particles incorporated into newly formed bone close to the original bone crest surface, 500 μm. Newly formed bone was also observed around particles further from the crest. In both images, residual membrane fragments were observed surrounding some graft particles. Acid fuchsin and toluidine blue stain.

However, residual particles were more frequently observed in more apical regions, in some cases beyond 6 mm from the CEJ (Figure 5a).

**Figure 6:**
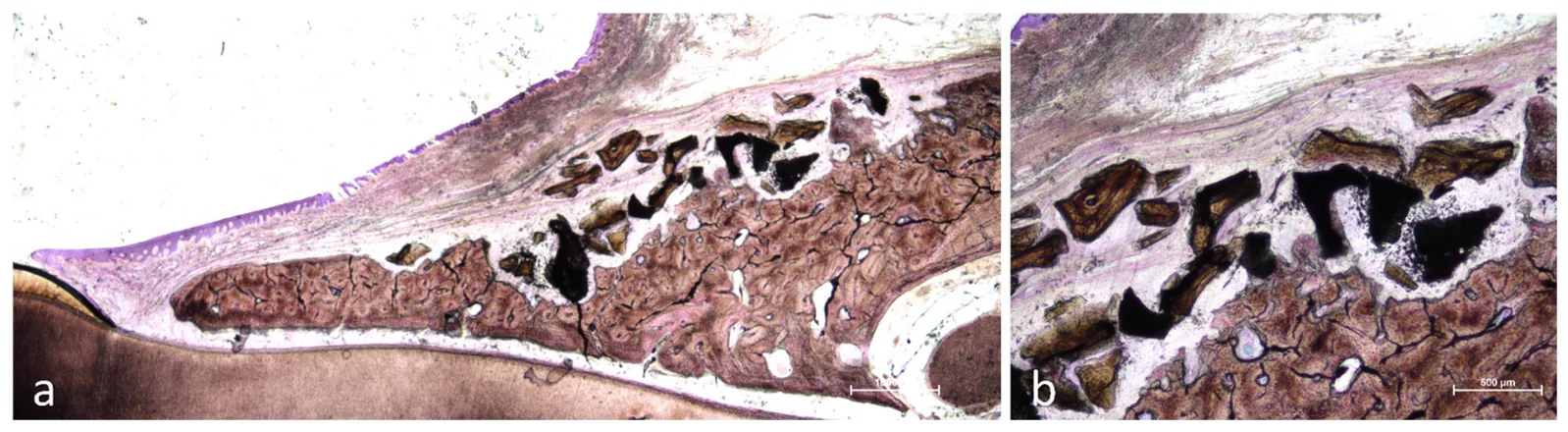
Photomicrographs of a ground section from the Pin group. (**a**) Residual graft particles were located mainly in more apical regions and appeared embedded in connective tissue rather than incorporated into newly formed bone, 1000 μm. (**b**) Higher magnification of the area shown in (**a**), illustrating residual graft particles surrounded by connective tissue without evident bone incorporation, 500 μm. In both images, residual membrane fragments were observed surrounding the graft particles. Acid fuchsin and toluidine blue stain.

In various specimens, residual graft particles appeared to be incorporated within newly formed bone (Figure 4b and Figure 5b), while in others they were mainly embedded in connective tissue (Figure 6a,b). Residual membrane fragments were also observed surrounding graft particles (e.g., Figure 5a and Figure 6a,b).

**Figure 7:**
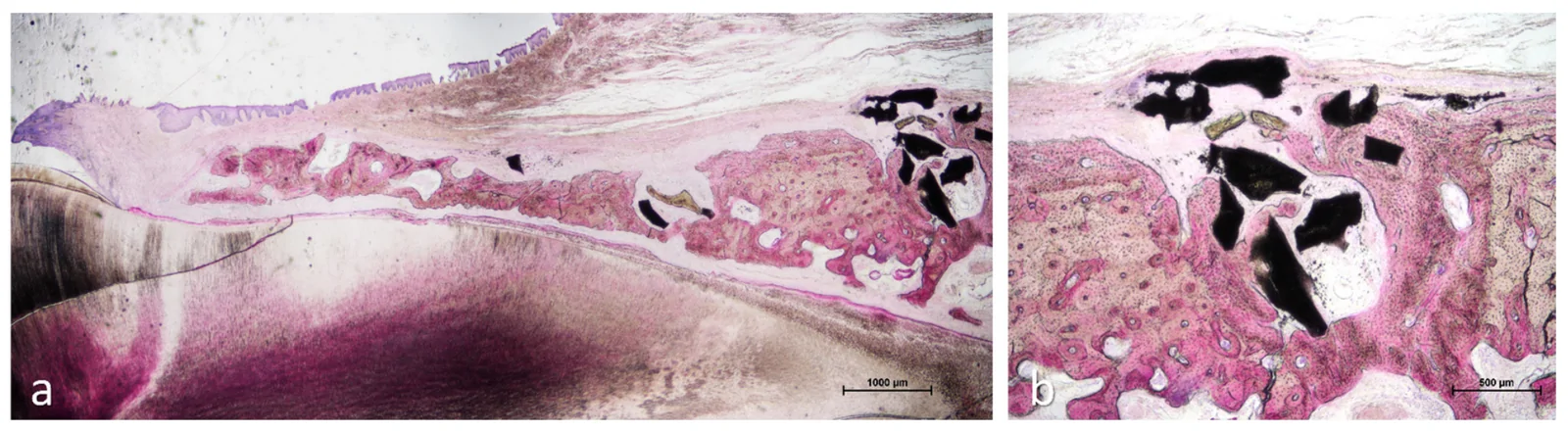
Photomicrographs of a ground section from the No-pin group. (**a**) Only a few residual graft particles were observed, mainly in apical regions. (**b**) Higher magnification of the area shown in (**a**), showing residual graft particles surrounded by connective tissue, with limited incorporation into newly formed bone. Acid fuchsin and toluidine blue stain.

In the No-pin group, only a few residual graft particles were identified (Figure 7a). These particles were generally sparse and mostly located away from the coronal portion of the ridge. Only occasional particles appeared to be incorporated within newly formed bone, whereas others were embedded in connective tissue (Figure 7b).

### 3.3. Quantitative Histological Evaluation

**Figure 8:**
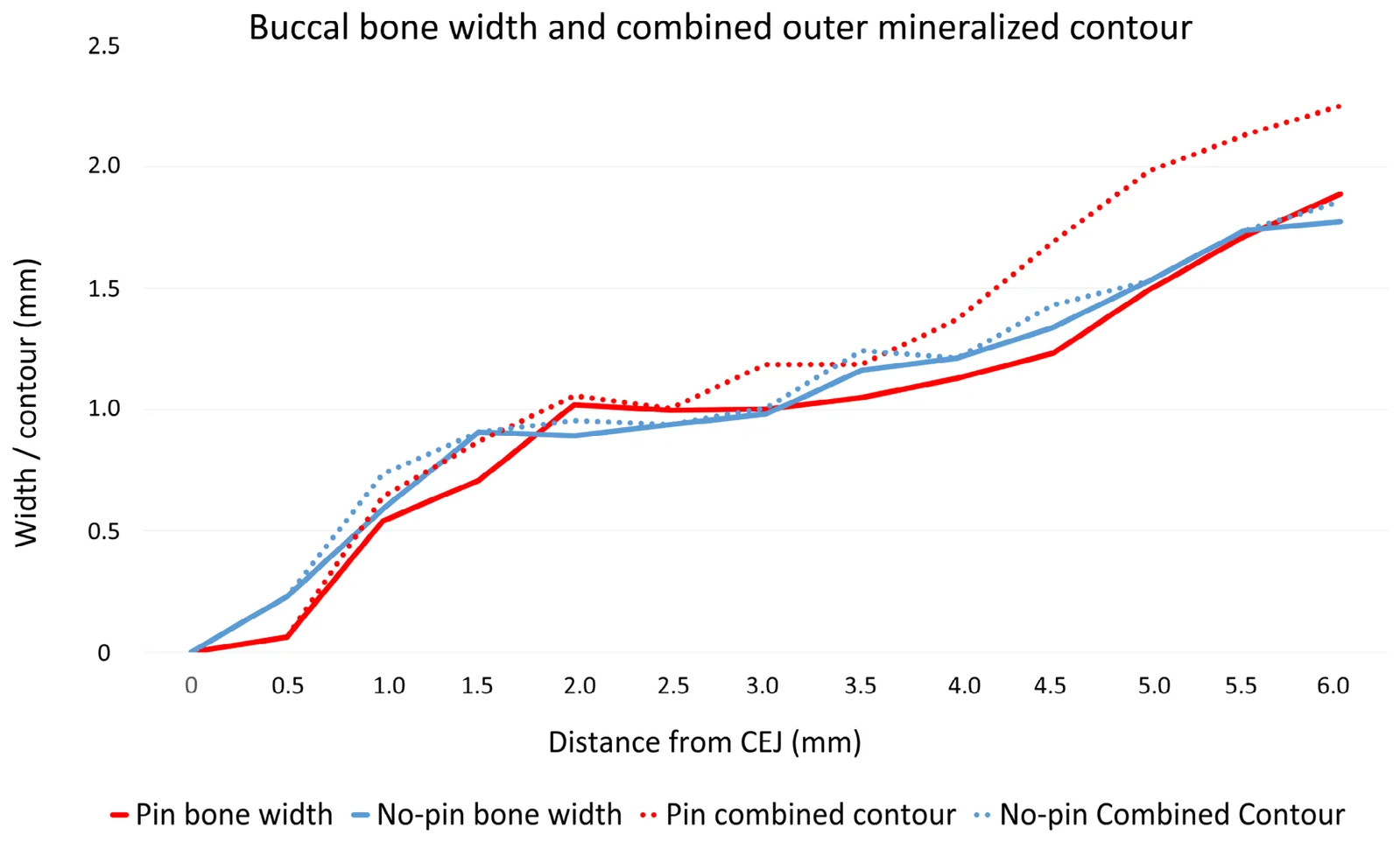
Horizontal buccal bone width and combined outer mineralized contour. Measurements were performed at the distal root using a lattice superimposed on the histological image, at 0.5-mm intervals from the cemento-enamel junction (CEJ) to 6 mm apically. Buccal bone width was measured from the inner margin of the alveolar crest to the outer bone contour. The combined outer mineralized contour (“Contour” in the graph) represents the outermost buccal limit of mineralized tissue, including residual graft particles only when they extended beyond the outer bone contour. In the No-pin group, the bone width and combined contour lines were almost superimposable, indicating a limited contribution of residual graft particles to the external buccal profile.

At 2 mm apical to the CEJ, the buccal bone width, measured from the inner margin of the alveolar crest to the outer bone contour, was 1.02 ± 0.30 mm in the Pin group and 0.89 ± 0.37 mm in the No-pin group (Figure 8; Table 1). The mean paired difference was 0.13 mm (95% CI, −0.55 to 0.81 mm; *p* = 0.6875), with a small standardized paired effect size (Cohen’s dz = 0.20).

At the same level, the combined outer mineralized contour measured 1.06 ± 0.37 mm in the Pin group and 0.95 ± 0.42 mm in the No-pin group. This secondary variable was evaluated descriptively and was not subjected to formal hypothesis testing.

The vertical distance from the CEJ to the most coronal level of the alveolar bone crest was 0.70 ± 0.32 mm in the Pin group and 0.61 ± 0.27 mm in the No-pin group. The most coronal level of the combined outer mineralized contour was located 0.69 ± 0.29 mm and 0.52 ± 0.23 mm apical to the CEJ in the Pin and No-pin groups, respectively. The complete horizontal profiles of bone width and combined outer mineralized contour are presented descriptively in Figure 8.

Within the standardized buccal region of interest extending from the alveolar crest to 6 mm apical to the CEJ, residual graft particles occupied 19.4 ± 6.9% of the analyzed area in the Pin group and 8.7 ± 6.6% in the No-pin group (Table 2). Bone occupied 69.6 ± 7.8% and 80.1 ± 10.6% of the analyzed area, respectively, whereas non-mineralized tissue occupied 11.0 ± 2.7% and 11.2 ± 6.4%. The non-mineralized tissue category included all tissue not classified as bone or residual graft and consisted predominantly of connective tissue surrounding the graft particles; marrow spaces were only occasionally present. Qualitatively, residual graft particles were also observed beyond the 6-mm reference level in some Pin specimens; these more apically located particles were not included in the histomorphometric region of interest.

## 4. Discussion

The present study evaluated whether fixation of a collagen membrane with titanium pins could influence buccal graft stability, graft containment, and the regional regenerative pattern in a pre-extraction buccal overbuilding model. The primary finding was that pin fixation did not significantly improve buccal bone width at 2 mm apical to the CEJ. The difference between groups was small and associated with a wide confidence interval, indicating considerable uncertainty regarding the magnitude of any potential effect. Thus, within the limits of this experimental model, pin fixation did not provide a measurable advantage in terms of coronal buccal bone augmentation. However, this should not be interpreted as a complete absence of biological effect, since qualitative observations indicated greater persistence of residual graft particles in the Pin group, particularly in more apical regions.

The combined outer mineralized contour, which represented the outermost mineralized profile including bone and residual graft particles, was numerically greater in the Pin group at 2 mm. However, this secondary outcome was evaluated descriptively and was not subjected to formal between-group hypothesis testing. The additional exploratory histomorphometric analysis showed a higher residual-graft fraction within the standardized buccal region in the Pin group than in the No-pin group, suggesting that fixation may have influenced graft retention even though it did not improve the primary coronal bone-width outcome.

The present findings are in line with the contradictory results reported in the literature regarding the additional benefit of membrane fixation in regenerative procedures [50,51,52,53] and are also consistent with previous experiments from our research group evaluating pre-extraction buccal overbuilding without pin fixation [44,45]. In one of these studies, pre-extraction buccal overbuilding did not preserve the alveolar bone crest dimensions after extraction and immediate implant placement; however, when the combined outer mineralized contour was considered, a greater volumetric gain was observed at the treated sites [44,45]. This distinction is relevant because pre-extraction buccal overbuilding may not serve only the preservation of the coronal buccal crest. Although its primary objective remains the support of the coronal buccal contour, maintenance of a more apical grafted compartment may also be relevant in selected prosthetically driven implant scenarios, where additional buccal support may be desirable in the apical portion of the future implant site.

Healing time may also influence the interpretation of these findings. In a previous similar study, buccal overbuilding with xenograft and collagen membrane without pin fixation resulted in more newly formed bone after 6 months than after 3 months, suggesting that a three-month healing period may not be sufficient for the xenograft to fully express its osteoconductive potential [45]. However, complete replacement of the graft particles by newly formed bone may not be essential for the rationale of this approach, particularly when the intended effect includes temporary space maintenance and preservation of a mineralized buccal compartment during subsequent healing phases.

The findings from the present study support the concept that membrane stability is only one of several factors influencing guided bone regeneration and ridge augmentation. Although fixation may reduce membrane mobility, the final regenerative outcome also depends on defect morphology, vascular supply, flap stability, graft particle stability, membrane degradation time, and the biological potential of the recipient site. In the present pre-extraction model, the graft was placed on the external surface of an intact alveolar ridge, without the socket or defect compartment that may contribute to clot and graft retention in conventional post-extraction or juxta-extraction ridge preservation. Therefore, the limited coronal effect observed in the present study should not be interpreted only as a failure of membrane fixation but rather as the consequence of a challenging non-contained model in which maintenance of a stable grafted compartment depends on multiple anatomical, mechanical, and biological factors. This interpretation is supported by a previous dog experiment in which immediate implants were placed in extraction sockets with standardized buccal defects treated with xenograft and collagen membrane, with or without pin fixation. In that study, no significant differences in new bone formation were detected between groups after three months, although the pin group showed greater retention of xenograft material and soft tissue [54]. Similarly, in the present study, membrane fixation did not significantly increase coronal bone dimensions, but the exploratory histomorphometric analysis suggested greater persistence of residual graft particles in the Pin group, supporting the possibility that fixation may influence regional graft containment even when this effect does not translate into a measurable improvement in coronal ridge augmentation.

The characteristics of the collagen membrane should also be considered when interpreting these findings. The membrane used in the present study was a native, non-chemically cross-linked collagen membrane derived from porcine pericardium. However, the degradation kinetics of native collagen membranes vary according to their tissue origin and structural organization. In vivo studies of pericardium-derived membranes have shown substantial degradation mainly between 8 and 12 weeks, with residual membrane still detectable at 12 weeks [55]. Therefore, the findings of the present study should not be attributed simply to complete membrane resorption during the early healing phase.

Nevertheless, the membrane was thin and flexible and did not possess the intrinsic rigidity required to maintain a stable three-dimensional space against soft-tissue pressure in a broad, non-contained defect. Pin fixation may have reduced membrane mobility but could not transform the collagen membrane into a self-supporting space-maintaining device. More rigid barriers, including non-resorbable PTFE membranes, titanium-reinforced devices, or cortical laminae, may provide greater structural support and graft containment in these conditions [16,17,18,19,20,21,22,23,24], although they are associated with different handling requirements and potential complications. Thus, the limited mechanical stability of the membrane-graft complex, rather than membrane degradation alone, may have contributed to the limited coronal augmentation observed in the present model.

In the present study, the exploratory histomorphometric analysis showed greater graft retention in the Pin group, whereas a lower amount of residual graft particles was identified in the No-pin group. However, these particles were not predominantly located in the coronal target region but were frequently observed in more apical portions of the buccal aspect. This distribution may be explained by the non-contained nature of the pre-extraction model and by the anatomical configuration of the mandibular molar region. In particular, the presence of the distal root limited ideal pin positioning and may have prevented direct compression and stabilization of the graft over the coronal target area. Consequently, fixation may have had a limited effect on coronal graft containment while still contributing to the persistence of the grafted compartment in a more apical position. Therefore, the apical presence of residual particles should not be interpreted only as undesirable displacement but also as part of the regional distribution pattern of the augmented compartment in this proof-of-principle model.

The extent and distribution of the grafted area may also have influenced the outcome. In the present model, the biomaterial was applied over a broad buccal surface in the mandibular molar region, where maintaining a stable and localized grafted compartment is technically challenging. Under these conditions, the final position of residual particles may not coincide with the coronal region selected as the primary outcome area, partly explaining the limited measurable effect on coronal ridge augmentation observed in both groups. This finding differs from previous pre-extraction studies in which graft particles were maintained in the experimental region despite the absence of pin fixation [43,44,45]. One possible explanation is the different anatomical region, since previous studies were performed in the premolar area, whereas the present experiment was conducted in the mandibular molar region. Differences in cortical bone thickness and density, soft-tissue thickness, flap mobility, and proximity of the masseter muscle may have influenced graft stability and healing, particularly in the absence of a contained defect. Therefore, the apical persistence of residual particles should not necessarily be regarded as biologically irrelevant but rather as a regional effect that may depend on graft distribution, anatomical conditions, and the intended target area. In this sense, the present model should be regarded as a proof-of-principle evaluation of graft containment and regional graft behavior rather than as a direct model of a specific clinical indication.

Besides anatomical differences, the type of graft may represent another relevant factor. In the present experiment, a bovine bone mineral fully sintered at 1200 °C was used, resulting in a highly crystalline, ceramic-like structure with a low resorption rate. Previous physicochemical analyses have shown that calcium phosphate-based bone substitutes may differ substantially from natural bone in terms of composition, crystallinity, and morphology [56]. Moreover, low-temperature processing of naturally derived bone grafts may better preserve collagen-related components and native physicochemical features, whereas high-temperature sintering alters crystallinity and produces a more ceramic-like structure [57]. Sintering temperature has also been shown to affect hydroxyapatite microstructure, porosity, mechanical properties, biodegradability/bioabsorbability, bioactivity, and biocompatibility [58], and similar considerations have been reported for bovine bone blocks processed at low and high temperatures for oral applications [59]. In the context of pre-extraction buccal overbuilding, these properties may have a dual interpretation: the low resorption rate may limit rapid replacement of graft particles by newly formed bone within a short healing period, but the persistence of a slowly resorbing mineral scaffold may be advantageous when the intended effect includes temporary space maintenance and preservation of a buccal mineralized compartment. Therefore, residual graft particles, particularly when associated with newly formed bone or maintained within the augmented area, should not necessarily be regarded as an unfavorable outcome but rather as one expected biological behavior of a slowly resorbing bovine bone mineral in this proof-of-principle model.

Another important consideration is that the procedure was performed before tooth extraction and therefore before the onset of extraction-related healing and remodeling. This design was selected intentionally to isolate the effect of membrane fixation on the formation and regional persistence of an augmented compartment on the external surface of an intact alveolar ridge. However, because tooth extraction and subsequent implant placement were not performed, the model did not reproduce post-extraction resorption or the healing and inflammatory environment associated with an extraction socket. Consequently, the presence of residual graft particles in more apical regions should be interpreted only as an exploratory observation concerning regional graft distribution. The present findings do not demonstrate that this distribution reduces the risk of buccal wall fenestration or perforation during subsequent implant placement. This possibility remains a hypothesis that should be evaluated in future studies specifically including tooth extraction, post-extraction healing, and implant placement.

The present findings indicate that pin fixation alone did not produce a measurable improvement in the coronal outcome in this non-contained model. The regional distribution of the residual graft may have been influenced by several interacting factors, including the initial graft position, local anatomy, membrane properties, and fixation. Further experimental studies are required to determine how these factors affect graft persistence after tooth extraction and whether the resulting regional distribution influences subsequent implant-site morphology.

Therefore, the predictability of pre-extraction buccal overbuilding may depend less on membrane fixation alone than on the interaction between graft distribution, fixation strategy, membrane rigidity, defect configuration, and the intended anatomical target. More space-maintaining barriers, different graft configurations, alternative fixation strategies, or more rigid reconstructive approaches may therefore be required to achieve stable and predictable buccal augmentation.

The present study has limitations. The sample size was limited, although a split-mouth design was used to reduce inter-animal variability and comply with the 3Rs principles for animal experimentation. The healing period was limited to three months, and longer observation periods may be needed to evaluate graft incorporation, particle persistence, and dimensional stability over time. In addition, only one type of particulate graft, one native collagen membrane, and one fixation approach were tested; therefore, the results may not be directly applicable to other biomaterials, membranes, or stabilization systems.

Another important limitation is that the model did not include tooth extraction and should therefore not be interpreted as direct evidence regarding post-extraction alveolar ridge preservation or subsequent implant placement. Moreover, the experiment was performed in the mandibular molar region, which does not reproduce the anatomical and prosthetic conditions of narrow anterior maxillary ridges. The possible relevance of the observed apical graft distribution to subsequent implant-site morphology remains unknown and requires evaluation in specifically designed studies including tooth extraction, post-extraction healing, and implant placement.

Despite these limitations, the study provides useful information on the biological behavior of pre-extraction buccal overbuilding and membrane stabilization on the external surface of an intact alveolar ridge. The absence of significant differences between fixed and non-fixed membranes suggests that membrane fixation, by itself, may not be a decisive factor in improving coronal buccal bone augmentation in this model. However, the exploratory histomorphometric analysis showed more residual graft particles in the Pin group and may indicate that fixation contributed to some degree of graft retention and regional persistence of the augmented compartment. Although this effect was not sufficient to significantly modify the primary histometric outcomes in the coronal region, it may be relevant for understanding how grafted compartments behave in non-contained pre-extraction conditions. Future studies should investigate whether more space-maintaining devices, more rigid barriers, alternative graft stabilization methods, or combined approaches may improve the predictability of pre-extraction or juxta-extraction buccal augmentation according to the intended anatomical region.

## 5. Conclusions

In conclusion, fixation of a collagen membrane with pins did not significantly improve buccal bone width, the prespecified primary outcome, compared with a non-fixed membrane in this pre-extraction buccal overbuilding model. The combined outer mineralized contour showed only small descriptive differences between groups. The exploratory histomorphometric analysis revealed a higher residual-graft fraction in the Pin group, although this outcome was not subjected to formal hypothesis testing. Thus, membrane fixation may have influenced graft retention but did not produce a measurable improvement in coronal buccal bone width after three months. The biological and clinical relevance of graft persistence remains to be determined in future studies including tooth extraction, post-extraction healing, and implant placement.

## Figures and Tables

**Table 1:** Horizontal and vertical measurements (mm) of bone and the combined outer mineralized contour.

Groups	Tissue/Contour	Horizontal Measurement at 2 mm Apical to CEJ (mm)	Vertical Distance from CEJ (mm)
Pin	Bone	1.02 ± 0.30	0.70 ± 0.32
	Combined outer mineralized contour	1.06 ± 0.37	0.69 ± 0.29
No-pin	Bone	0.89 ± 0.37	0.61 ± 0.27
	Combined outer mineralized contour	0.95 ± 0.42	0.52 ± 0.23

**Table 2:** Percentages of bone, residual graft, and non-mineralized tissue within the standardized buccal region of interest.

Groups	Bone%	Graft%	Non-Mineralized Tissue%
Pin	69.6 ± 7.8%	19.4 ± 6.9%	11.0 ± 2.7%
No-pin	80.1 ± 10.6%	8.7 ± 6.6%	11.2 ± 6.4%

## Data Availability

Dataset available on request from the authors.
